# Prevention of Tendon Subluxation in Dequervain’s Tenosynovitis Release Using Retinacular Repair

**DOI:** 10.51894/001c.4705

**Published:** 2016-10-24

**Authors:** Brandon J. Horn, Robert Zondervan, Gail Shafer-Crane, Erich Hornbach

**Affiliations:** 1 Department of Orthopedic Surgery McLaren-Greater Lansing Hospital; 2 Division of Human Anatomy Michigan State University

**Keywords:** dequervain’s tenosynovitis, surgery

## Abstract

This study compared the incidence of tendon subluxation in patients of a single surgeon undergoing Dequervain’s release with and without retinacular repair. The study reviewed 31 patients that underwent standard Dequervain’s release without retinacular repair and 49 that underwent Dequervain’s release with retinacular repair. Each subject’s functional status was assessed using the Patient-Rated Wrist/Hand Evaluation. Subjects were compared against age, gender, handedness, tendon subluxation, return to work duration, and surgical laterality. Tendon subluxation is an infrequent complication affecting patients undergoing Dequervain’s release. This complication has a higher incidence in younger females and demonstrates no predilection for hand dominance. The efficacy of retinacular repair is suggested by good Patient-Rated Wrist/Hand Evaluation outcome scores and should be considered as an adjunct to prevent tendon subluxation. This is a level 4 study.

## INTRODUCTION

Dequervain’s disease is characterized by stenosis tenovaginitis of the first dorsal compartment of the wrist. It is a debilitating disease affecting both males and females, but with a significantly higher prevalence in the female population.^1^ The incidence of Dequervain’s tenosynovitis has been studied and reported to have a range of 0.31-0.94 per 1000.^1–3^ A study using the working population in the Loire region of France segregated Dequervain’s tenosynovitis as a specific diagnosis and reported that over 2 years incidence of Dequervain’s tenosynovitis was found to be 12 per 1000, with 2.1% of females presenting with this diagnosis, compared to 0.7% (11/1,566) men.^4^ For all four of the reported studies, the diagnosis of Dequervain’s tenosynovitis included pain and inflammation of the first dorsal compartment tendons.

Symptomatic relief from Dequervain’s disease has been attempted through conservative and invasive treatments.^5^ After failing conservative attempts, surgical procedures have historically involved release of the first dorsal compartment and septum, followed by debridement of synovitis.^5^ However, this procedure is not without risk, and complications include nerve injury, tendon subluxation, and inadequate release of the compartment.^6^

The complication of tendon subluxation after surgical release of Dequervain’s disease has only been described in case reports.^7–9^ Prevention of the complication has been attempted intraoperatively by releasing the first dorsal compartment sheath along its dorsal margin, thus leaving the volar flap intact to prevent subluxation of the tendon.^10^ Alternatively, z-plasty of the first dorsal compartment has been described as a preventive measure.^11^

Despite these preventive techniques, the senior author (E.H.) encountered 12 cases of tendon subluxation after Dequervain’s release during a 10-year period. The complication was observed in patients within his practice and from referral. In all 12 cases that were observed, the patients reported painful tendon subluxation and limited hand dexterity, necessitating revision surgery.

Tendon subluxation is surgically treated through a brachioradialis flap^8^ and/or an extensor retinaculum reconstruction.^7^ While these restorative treatments have been shown to have positive outcomes, efforts should be focused on the prevention of tendon subluxation from Dequervain’s release.

For the purposes of this study, it was hypothesized that patients undergoing Dequervain’s release that included preventive retinacular repair would have a decreased incidence of tendon subluxation. This hypothesis was tested through a retrospective review of the primary author’s cases of patients undergoing Dequervain’s release without retinacular repair and those undergoing a described technique for retinacular repair.^12^ A decrease in tendon subluxation incidence in the retinacular repair population will support the adoption of this novel technique to reduce Dequervain’s release complications.

## METHODS AND TECHNIQUES

Approval from the Michigan State University Institutional Review Board was obtained before undertaking this study (IRB# 14-430 Category: EXPEDITED 5, 7; approval date May 14, 2014). A retrospective review was conducted to identify patients who underwent Dequervain’s release by a single surgeon during a 10-year period (May 2002 to November 2013). Patients were included in the study if they underwent a Dequervain’s release and met a minimum follow up time of at least 6 months. A total of 80 patients were identified. Of those, there were 31 that underwent Dequervain’s release without retinacular repair and 49 underwent Dequervain’s release with retinacular repair. Subjects were excluded from our study if they were lost to follow up and functional status was not assessed using the Patient-Rated Wrist/Hand Evaluation.

Patients qualified for surgery based on physical exam findings of tenderness along the first dorsal compartment tendon sheath and positive Finkelstein exam. Patients were also given two serial steroid injections prior to surgery and conservative management attempted. Conservative management included activity modification and prescription NSAID trial. If they failed conservative management, they were taken for Dequervain’s release without or with retinacular repair. All patients underwent the same procedure described in the surgical technique described below with variable repair of the extensor retinaculum. They were seen in follow up at 2 weeks, 4 weeks, 6 weeks, and 6 months. Every patient underwent the same postoperative protocol described below.

Patients that underwent Dequervain’s release with retinacular repair underwent a subsequent phone interview between 6/1/14 and 6/7/14. Data were collected on patient handedness, return to work, and a PRWHE test was verbally administered to assess pain and function of the wrist.

**Figure 1:**
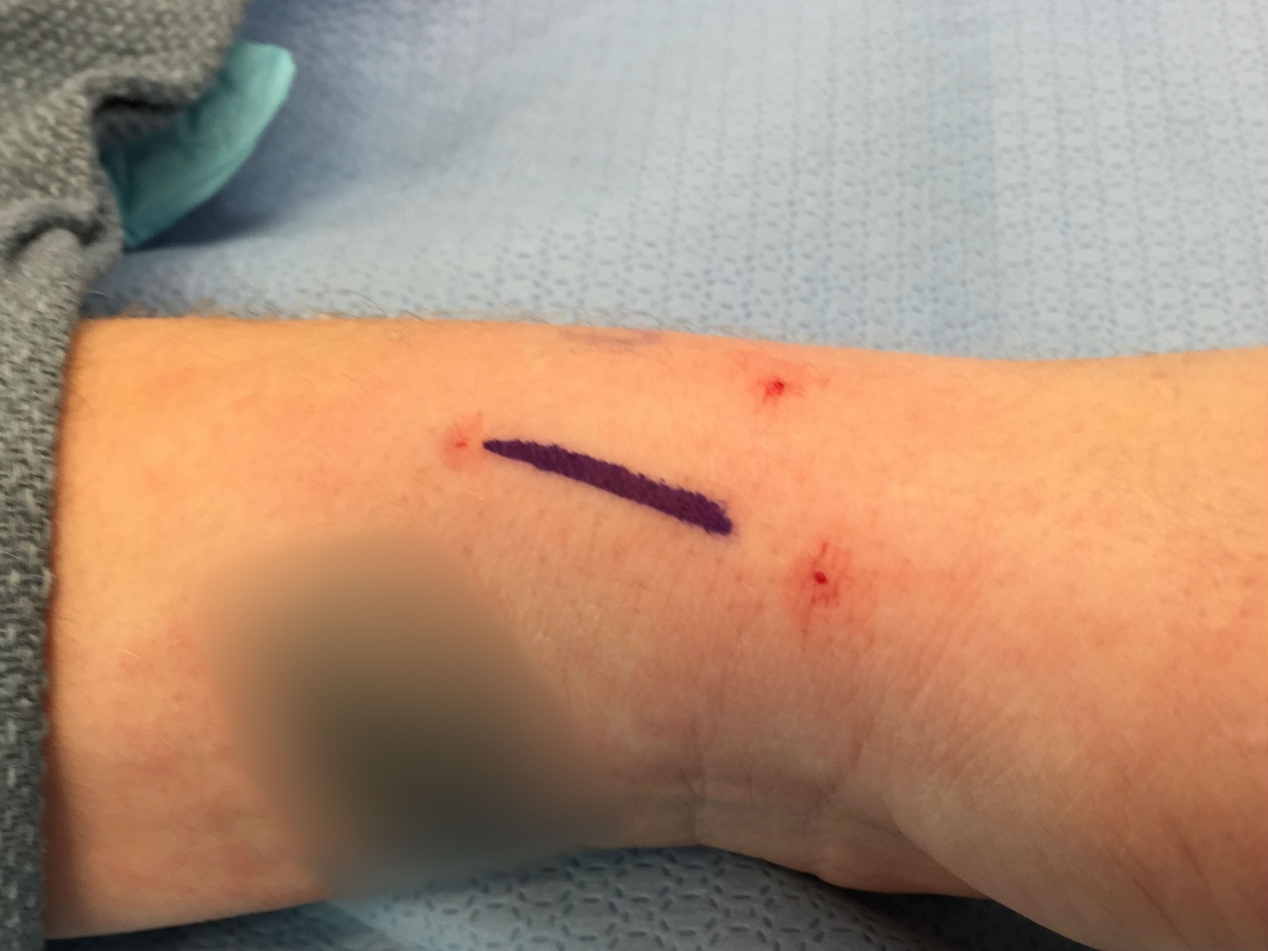
Demonstration of the longitudinal skin incision for Dequervain’s release.

**Figure 2:**
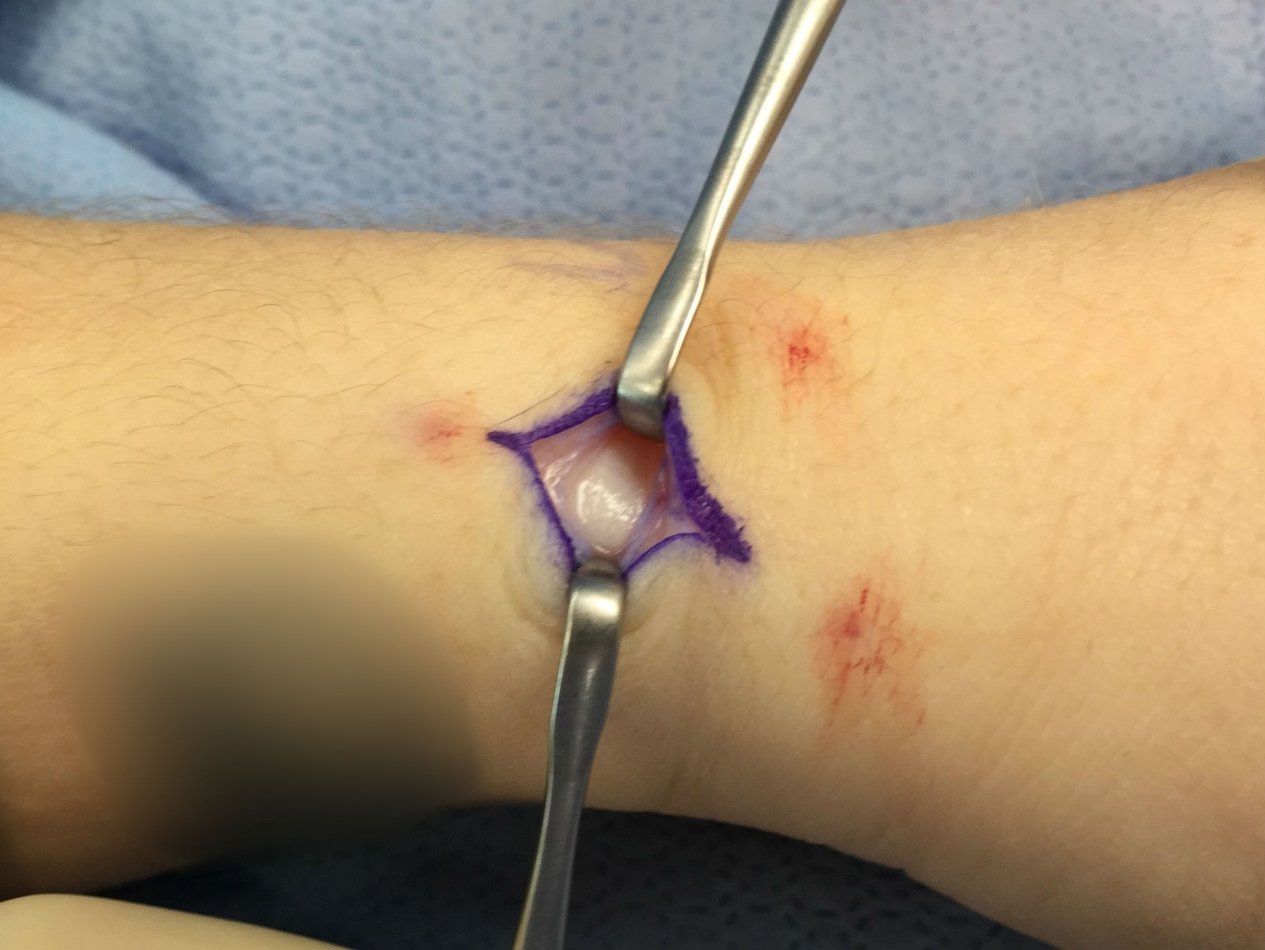
Demonstration of the incision in the extensor retinaculum between the volar and dorsal 2/3 of the first dorsal compartment longitudinally.

*SURGICAL TECHNIQUE FOR RETINACULAR REPAIR.* Dequervain’s release is performed under monitored anesthesia care with local infiltration of 1% lidocaine. A proximal arm tourniquet is used and longitudinal skin incision is made beginning 1 cm proximal to the radial styloid and extending proximally along the dorsoradial aspect of the distal forearm for 3 to 4 cm (Figure 1). Branches of the radial sensory nerve are identified by gentle-blunt transverse dissection. The extensor retinaculum is incised between the volar and dorsal 2/3 of first dorsal compartment longitudinally (Figure 2).

**Figure 3:**
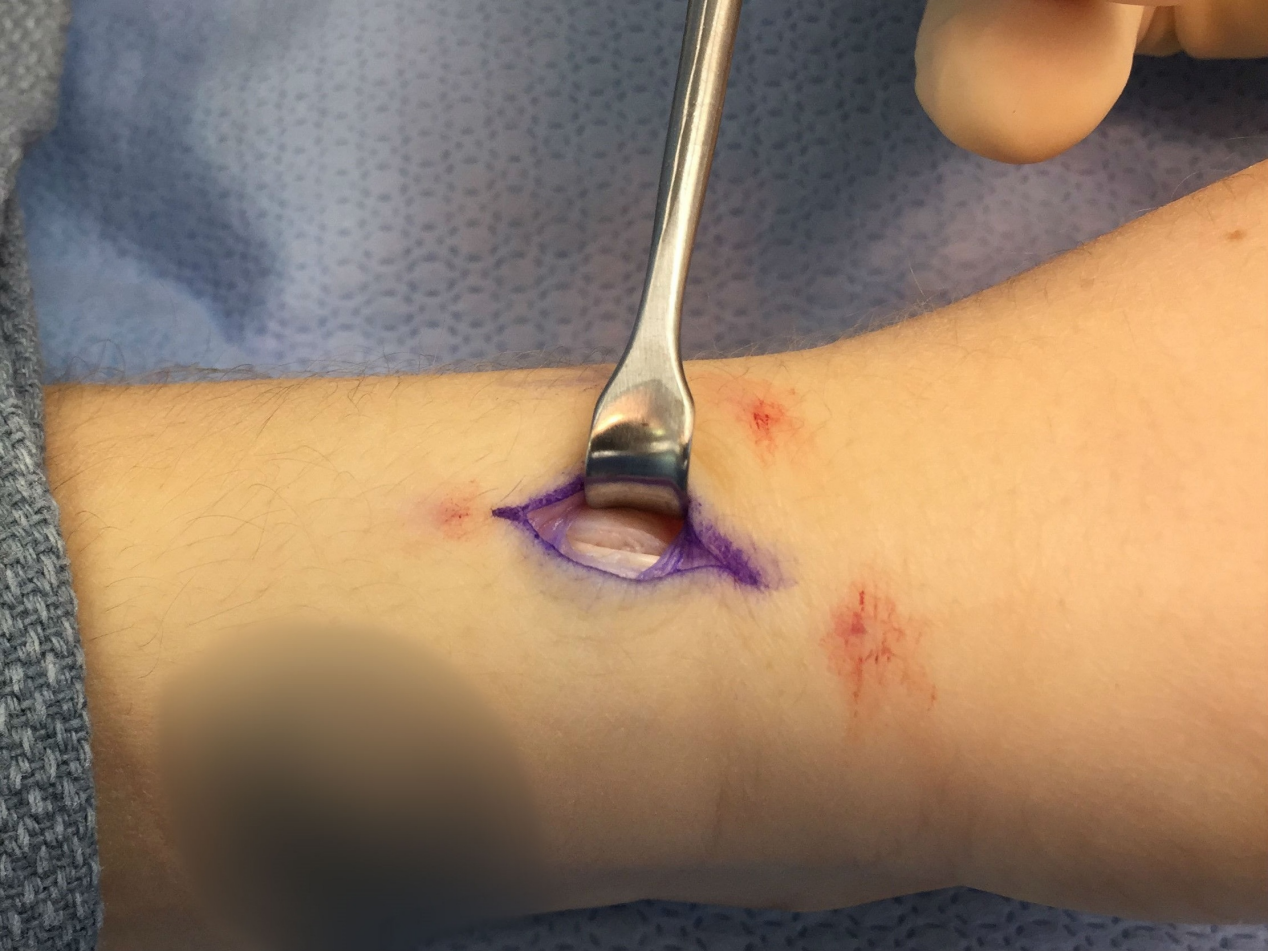
Demonstration of removal of excess synovium from the tendon sheaths.

The first dorsal compartment is carefully explored for the tendon of the extensor pollicis brevis (EPB), and the multiple tendon slips of the abductor pollicis longus (APL). The fibroosseous canal is examined for septation and extra aberrant tendons. After exploration and retraction of EPB, a search is made volarly for an intra-compartmental septum and removed if present. Synovectomy is performed along each of the tendon sheaths (Figure 3). At this point, if no retinacular repair is performed, incision site skin edges are approximated and closed using 5-0 nylon. In patients undergoing retinacular repair, the Idler technique is implemented.^12^

**Figure 4:**
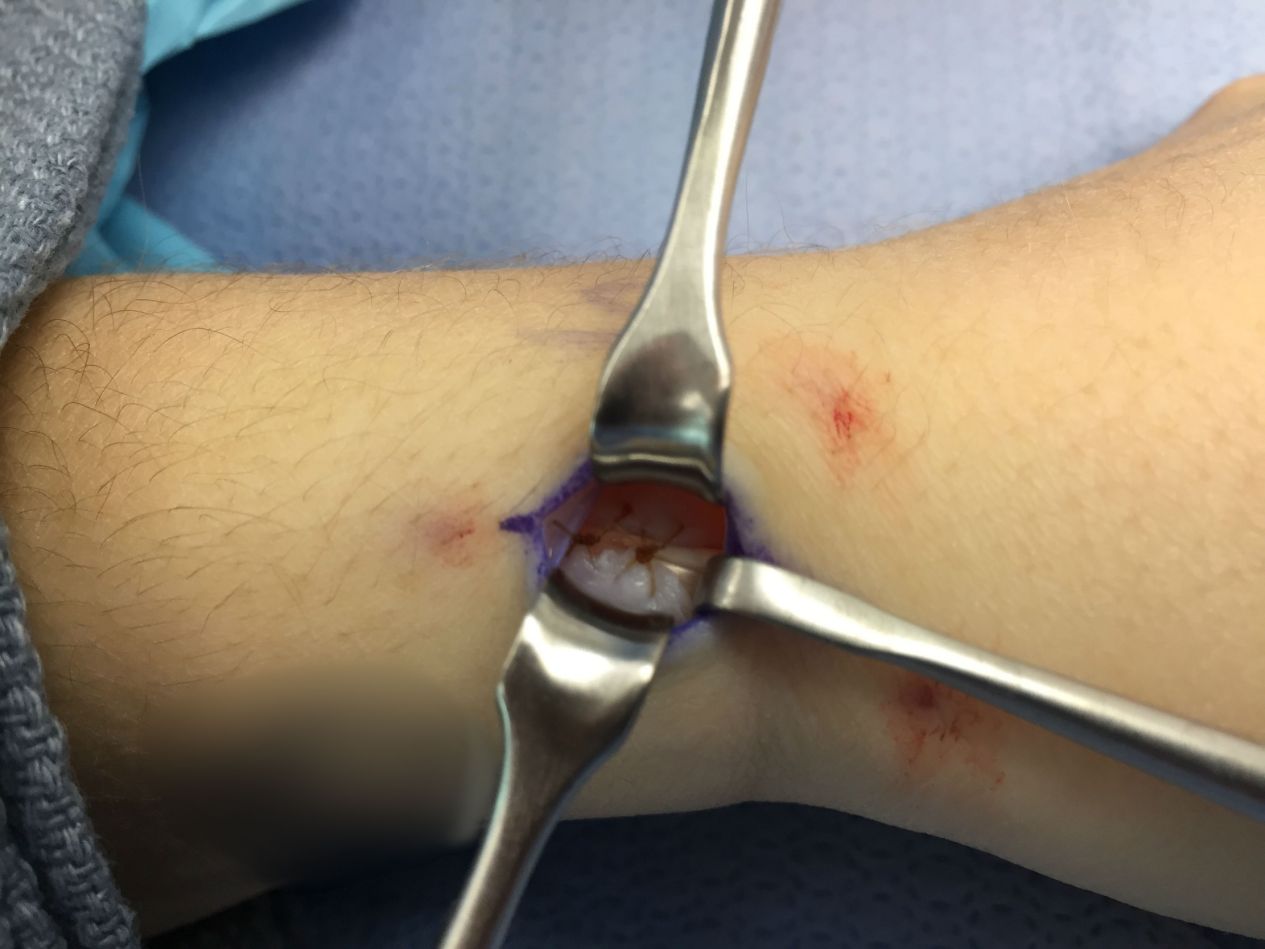
Demonstration of the retinacular repair with 3-4 mm gap.

**Figure 5:**
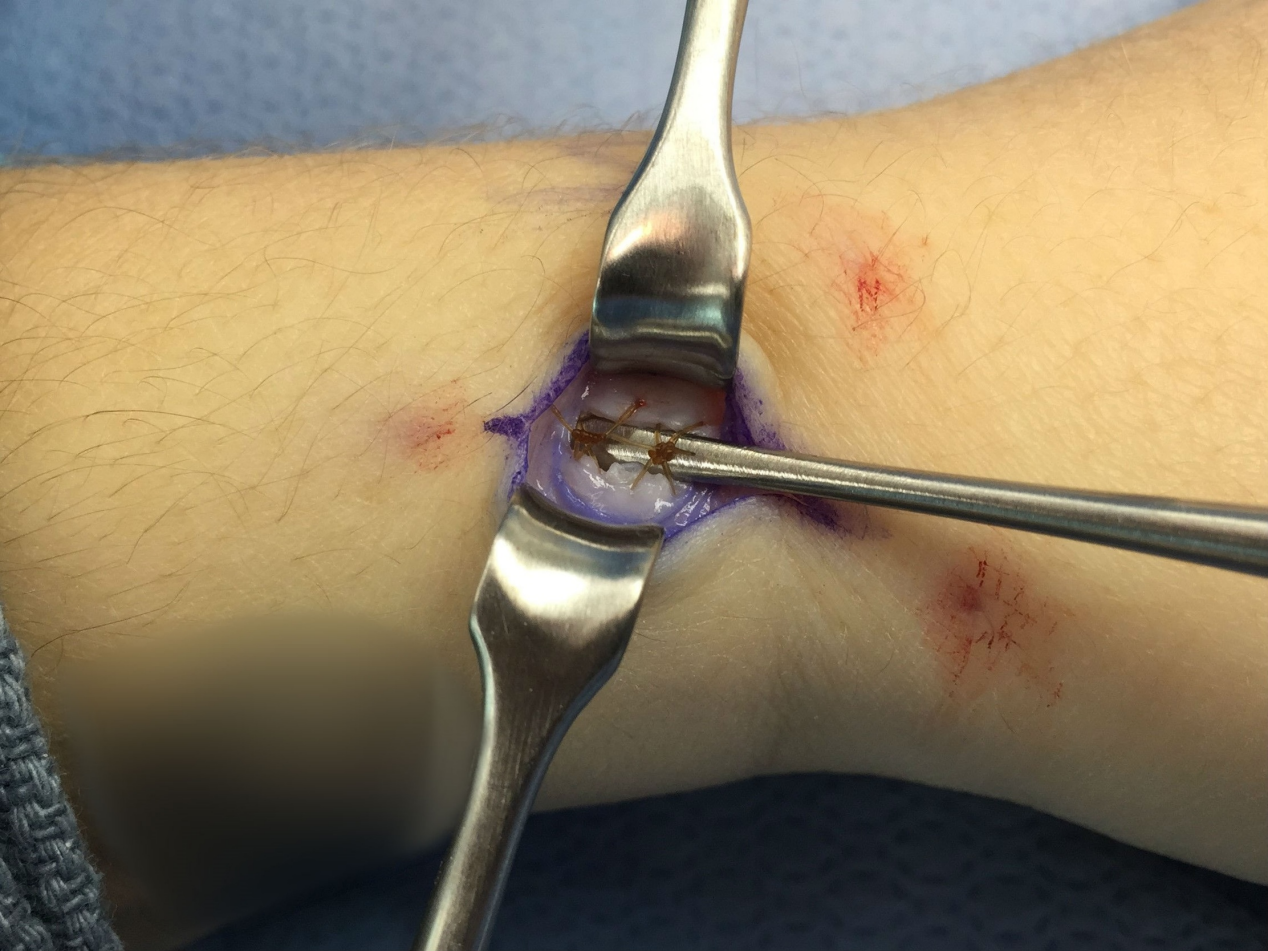
Demonstration of testing of tension of repair performed by rotating a Freer elevator 360 degrees under the retinacular flap.

4-0 chromic simple stitches are used to approximate the volar and dorsal retinacular flaps with a 3-4 mm gap (Figure 4). Testing of tension of repair is performed by rotating a Freer elevator 360 degrees under the retinacular flap, if unable to completely rotate the retractor, then repair is reperformed (Figure 5). Skin incisions are subsequently approximated and closed with 5-0 nylon. Sterile dressings and a thumb spica splint are applied.

*POST OPERATIVE PROTOCOL.* Post operatively the patients are kept in thumb spica splint for 2 weeks until seen at the 2-week visit. Patients are then placed in a removable thumb spica splint. The patient is instructed to remove the splint 6 times a day to allow thumb ulnar deviation to 40 degrees and 20 degrees flexion/extension for the first two weeks. During the third and fourth weeks, patients are instructed to remove the splint six times per day and preform gentle circumduction. Range of motion is increased to maximum ulnar deviation at that time. Thumb radial deviation is encouraged during the fifth and sixth week and light loading of the wrist is allowed with interval removal of the thumb spica. After six weeks, the patients are allowed to return to full activity and thumb spica is completely removed. This is also the point that full duty is allowed at work.

*STATISTICS.* Chart reviews and telephone interviews were conducted for data collection. The goals of this study were to compare age, gender, and identify tendon subluxation between study groups. The group undergoing retinacular repair was also assessed with Patient-Rated Wrist/Hand Evaluation (PRWHE) and identified for handedness, surgical laterality, and duration for return to work.

Study data is presented as values with percentages for categorical variables or mean values ± standard deviation for continuous variables. Comparison of age between study groups was made using Student’s t-test. Comparisons of categorical variables (gender and tendon subluxation) were made using Fischer’s exact test. In the revision population, descriptive statistics were calculated for PRWHE and return to work. Handedness and surgical laterality were compared using Fisher’s exact test. For all statistical tests the level of significance was set at *p* < 0.05. Statistical analyses were performed using MATLAB 7.12 R2011a (Natick, MA).

## RESULTS

Of the 80 patients included in the study, 39% (31/80) underwent Dequervain’s release without retinacular repair and 61% (49/80) underwent Dequervain’s release with retinacular repair (Table 1). The average age overall for the study population was 55.6 ± 13.4. The average age for patients undergoing Dequervain’s release without and with repair was 47.8 ± 12.8 and 60.9 ± 11.2, respectively. The patients with repair were significantly older than those without (*p* < 0.001).

**Table 1.** Population Demographics and Tendon Subluxation Complications.

	**Total**		**Without Repair**			**With Repair**			
**Variable**								**p**	**Test**
**n (%)**	80 (100%)		31 (38.8%)			49 (61.3%)		**-**	**-**
**Mean Age, *years* (SD)**	55.6 (±13.4)		47.8 (±12.8)			60.9(±11.2)		<.001	Student's
**Male (%)**	5 (6.3%)		1 (3.2%)			4 (8.2%)		0.35	Fischer
**Subluxation (%)**	2 (2.5%)		2 (4.2%)			0 (0.0%)		0.47	Fischer

Comparisons of age, gender, and tendon subluxation between patients undergoing Dequervain’s release without and with retinacular repair.

The overall study population was comprised primarily of females with males only accounting for 3.2% (1/31) and 8.2%(4/49) of the populations without and with retinacular repair, respectively. No significant gender difference was identified between the two study groups (*p* = 0.35).

Tendon subluxation was identified in 2.5% (2/31) of the patients without retinacular repair. This complication was not observed in patients with retinacular repair. However, the difference between the two populations did not reach statistical significance (*p* = 0.47).

For the retinacular repair population, PRWHE scores, handedness, and return to work are described in Table 2. In this population 44.4% (20/45) received surgery ipsilateral to the dominant hand, 41.5% (17/41) and 75% (3/4) in right and left hand dominant patients, respectively. The difference between surgical laterality and handedness did not reach statistical significance (*p* = 0.31) (Table 3).

**Table 2.** Retinacular Repair Follow-up Patient Data.

	**With Repair**
**Variable**	
Mean PRWE ^‡^ (SD)	1.93 (±3.85)
Right Handedness (%)	41 (91%)
Return To Work (%)	
*When released*	14 (31%)
*With restrictions*	14 (31%)
*Retired*	17 (38%)

Data from phone interviews status post retinacular repair. Four of the original 49 patients were lost to follow-up and not included in this data.

^‡^Patient rated wrist evaluation (PRWE) is a questionnaire to measure wrist pain and disability. It is scored out of 100 with 0 being the best outcome.

**Table 3:** 

	**Handedness**		
**Surgical Site**	**RHD**	**LHD**	**Total**
**Ipsilateral**	17	3	20
**Contralateral**	24	1	25
**Total**	41	4	**45**
			
		**p =**	**0.31**

Surgical Laterality and Patient Handedness in the Retinacular Repair Group

## DISCUSSION

This study investigated two distinct populations of patients who underwent surgical treatment for Dequervain’s tenosynovitis. In the patient population that underwent a Dequervain’s release without retinacular repair, two cases of tendon subluxation were observed. Both patients had painful symptomatic tendon subluxations and required revision reconstruction of the first dorsal compartment. Patients that were in the second group that underwent a Dequervain’s release with retinacular repair had no reports of tendon subluxation. It is important to note that although this difference did not reach statistical significance, it suggests retinacular repair may be a viable preventive measure, and warrants further study. Given the study’s incidence of subluxation, a sample population of 364 patients would be needed (182 in each treatment group) to detect a statistical difference with a power of 80%. Considering the 80 Dequervain’s release procedures that were included in this study took over 8 years to collect, it would take approximately 35 years to accrue the required sample size. The time needed to create such a large sample size for a single surgeon in a specific procedure would be prohibitively long, which supports future studies of Dequervain’s tenosynovitis using multiple sites/surgeons.

Even though the complication of tendon subluxation is rare and has only been identified in case reports, patient’s that do present with this complication suffer from pain and loss of thumb function with tendon subluxation and ultimately require revision surgery. The addition of retinacular repair by the senior author (E.H.) was seen as a proactive measure, which requires minimal additional time or effort, yet has the potential to prevent subluxation.

Our study identified gender as a significant factor in the occurrence of Dequervain’s tenosynovitis. When combining those without and with retinacular repair, 94% (75/80) were female (*p* < 0.05). This is consistent with the results seen by Wolf et al. who reported women having a significantly higher rate of Dequervain’s tenosynovitis at 2.8 cases per 1000 person-years, compared to men at 0.6 per 1000 person-years.^1^ Age greater than 40 also was a significant risk factor, with this age category showing a rate of 2.0 per 1000 person-years compared to 0.6 per 1000 in people under 20 years of age. Although assessment of age as a risk factor cannot be performed with our population data, our patient average age (56.6 ± 13.4) is consistent with the previously reported age of greater risk. This supports increased age and work demands as possibly associated with an increased occurrence of Dequervain’s tenosynovitis.

Limitations to this study are that it is not prospective and randomized. Patient demographics between groups were partially matched, with a significant difference in age between groups. Another limitation to the study was the follow up of 6 months; we were not able to confirm that tendon subluxation did not occur after 6 months. However, tendon subluxation that has been described occurred within 1-2 months of surgery, and not later.^8^ The two patients from this study that presented with subluxation had symptoms within the first month after surgery.

Hand dominance has not been previously identified as a factor causing Dequervain’s tenosynovitis.^1^ Our data also shows that hand dominance is not a factor in predicting operative side. However, we did observe an increased incidence of Dequervain’s tenosynovitis in the contralateral hand of RHD patients, while in LHD patients the incidence was higher in the ipsilateral hand, an unexplained finding that warrants further investigation.

Following Dequervain’s release with retinacular repair, PRWHE assessments demonstrate minimal functional limitations and minimal pain with associated activities. This is important because although our results for the prevention of tendon subluxation with repair do not reach statistical significance, no patients in the repair group experienced subluxation. This study suggests Dequervain’s release with retinacular repair may be a viable preventive measure for tendon subluxation and to reduce the need for repeat surgery.

### Conflict of Interest

The authors declare no conflict of interest.
